# Equal status in Ultimatum Games promotes rational sharing

**DOI:** 10.1038/s41598-018-19503-x

**Published:** 2018-01-19

**Authors:** Xiao Han, Shinan Cao, Jian-Zhang Bao, Wen-Xu Wang, Boyu Zhang, Zi-You Gao, Angel Sánchez

**Affiliations:** 1 MOE Key Laboratory for Urban Transportation Complex Systems Theory and Technology, Beijing Jiaotong University, Beijing, 100044 P. R. China; 2 School of Systems Science, Beijing Normal University, Beijing, 100875 P. R. China; 3 School of Finance, University of International Business and Economics, Beijing, 100029 P. R. China; 4 Laboratory of Mathematics and Complex Systems, Ministry of Education, School of Mathematical Sciences, Beijing Normal University, Beijing, 100875 P. R. China; 5 Grupo Interdisciplinar de Sistemas Complejos (GISC), Departamento de Matemáticas, Universidad Carlos III de Madrid, 28911 Leganés, Spain; 6 UC3M-BS Institute of Financial Big Data (IFIBID), Universidad Carlos III de Madrid, 28911 Leganés, Spain; 7 Instituto de Biocomputación y Física de Sistemas Complejos (BIFI), Universidad de Zaragoza, Zaragoza, Spain; 8 Unidad Mixta Interdisciplinar de Comportamiento y Complejidad Social (UICCS) UC3M-UV-UZ, Universidad Carlos III de Madrid, 28911 Leganés, Spain

## Abstract

Experiments on the Ultimatum Game (UG) repeatedly show that people’s behaviour is far from rational. In UG experiments, a subject proposes how to divide a pot and the other can accept or reject the proposal, in which case both lose everything. While rational people would offer and accept the minimum possible amount, in experiments low offers are often rejected and offers are typically larger than the minimum, and even fair. Several theoretical works have proposed that these results may arise evolutionarily when subjects act in both roles and there is a fixed interaction structure in the population specifying who plays with whom. We report the first experiments on structured UG with subjects playing simultaneously both roles. We observe that acceptance levels of responders approach rationality and proposers accommodate their offers to their environment. More precisely, subjects keep low acceptance levels all the time, but as proposers they follow a best-response-like approach to choose their offers. We thus find that status equality promotes rational sharing while the influence of structure leads to fairer offers compared to well-mixed populations. Our results are far from what is observed in single-role UG experiments and largely different from available predictions based on evolutionary game theory.

## Introduction

The Ultimatum Game (UG) was proposed more than three decades ago^1,2^ as a simple and clear way to measure social preferences^3^. In UG experiments, experimenters work with two subjects and give one of them (the “proposer”) an amount of money. The proposer makes an offer as to how to split the money to the other player (the “responder”). The responder can only accept the proposal as is or reject it outright, and in case of rejection none of the players receives any money. Clearly, rational people, where the term “rational” is used in the sense of self-interest, will both offer and accept the minimum possible amount, as responders have no incentives to reject any positive amount of money. However, all available experiments provide strong evidence that low offers are often rejected, with low meaning lower than 20–30% of the pot. Correspondingly, it appears that proposers anticipate this behaviour and offer amounts larger than the minimum, with fair splits being frequent. It is worth stressing that in the last three decades literally thousands of experiments have been carried out^4–9^ giving the same qualitative results.

A common variant of the standard ultimatum game is that the responder precommits a minimum acceptable offer (MAO) that he/she will accept (and any lower offer will be rejected) rather than simply decides whether to accept a specific offer. This MAO variant is more informative about responders’ preferences. Experiments found that the minimum acceptable offers for most subjects are around 30%^6,10–12^. This is consistent with the observations of the standard UG that offers lower than 30% are often rejected^8,9^. In particular, the MAO UG has some relation with the coordination game in the sense that when the proposer’s offer equals to the responder’s acceptance level, then both of them has no incentive to change their strategies^13^.

These clear, reproducible experimental results have puzzled economists, but also evolutionarily biologists for a long time. Indeed, the fact that human subjects reject positive amounts of money out of anger about what is considered to be unfair is hard to reconcile with the self-interested decisions one would expect from evolutionarily selected species. In this respect, it is interesting to note that this behaviour has been also observed among non-human primates^14^. Therefore, a number of explanations from different disciplines and perspectives have been proposed in order to understand the reasons for this behaviour. Prominent among these are theoretical approaches based on evolutionary game theory^10,15–25^. Most of these studies considered a variant of standard UG, where subjects play both roles and their strategies are given by two parameters *p* and *q*. When acting as proposer, the player offers the amount *p*. When acting as responder, the player rejects any offer smaller than *q*. Thus, in this variant, subjects play both roles in a MAO UG. Similarly to the standard UG, a rational responder should accept any (non-zero) offer, and therefore, a rational proposer will offer the minimum. It is worth noting that if everyone follows the same strategy in a dual-role UG, then all the subjects will obtain the same payoff, which is a fair outcome. In this paper, we define fairness as fair share in order to keep consistent with previous theoretical and empirical studies.

While these evolutionary game-theoretical models touch upon different aspects of the problem, two factors have been identified in the literature as possibly relevant for the arising of quasi-fair offers and the rejection of unfair offers: First, subjects taking part in the UG in both roles, meaning that they have to be both proposers and responders. Second, subjects interacting with fixed partners during the repeated play (i.e., there is a fixed interaction structure)^16,19,21–25^. In other words, evolutionary game theory on a well-mixed population where everybody plays with everybody else predicts convergence to self-interested behaviour, and only when interactions are restricted to a few, fixed members of the population non-zero offers and acceptance levels arise.

In this paper, we experimentally test the above two factors. Our dual-role repeated UG experiments cover three key issues hitherto unaddressed in the literature. First, it allows for direct comparison to the theoretical predictions. The starting point of most theoretical research based on evolutionary game theory is the dual-role UG: agents act in both roles, and their choices for the acceptance level, *q*, and the offer, *p*, evolve with different prescriptions based on their and other subjects’ payoffs and/or actions^10,15–25^. We note that some of these studies assumed that players are equally likely to be in either of the two roles and, in this case, the expected payoff of a player is half of that earned when acting in both roles^15–17^. However, in the midst of the abundant experimental literature on the UG, only a few studies consider a setup given by a dual-role UG. These studies typically resort to the strategy method: subjects pick the two values and then they are randomly matched in a specific role in which the game is realized^4,26–30^. Second, to our knowledge, all the available experiments on the dual-role UG are one-shot and have not provided a clear picture of the behaviour in dual-role UGs: Carter and Irons^26^ and Weg and Smith^27^ found that proposer demands were greater if subjects play both roles. Conversely, Güth and Tietz^4^ found that proposers who play both roles make smaller demands than those who do not. Third, aside from an experiment on a bipartite structure of proposers and responders, the effects of population structures on UG experiments are unclear^31^. Although several theoretical studies indicated that a fixed interaction structure can enhance fairness^16,19,21–25^, this point has not been tested by laboratory experiments. In addition, a structure setting allows us to get insight into how subjects decide their actions given the information about their neighbours’ behaviours in the previous round. Finally, a word is in order about the connection of this work with real contexts. While we do neither claim nor believe that there are actual, specific systems or situations that can be modeled by our experimental setup (or by the corresponding theoretical models for that matter), we do think that the mechanisms we are exploring will indeed be relevant to many such situations in order to provide possible explanations of the observed behaviours.

## Results

We conducted a series of repeated dual-role UG experiments to test the effects of playing both roles and population structures on UG. The experiments include 9 treatment groups T1-T9 with structured populations and 2 control groups C1-C2 with well-mixed populations (see Methods, Supplementary Notes 1.1–1.3, Supplementary Table 1 and Supplementary Figures 1–3 for more details). In the 9 treatment groups, every subject plays with four fixed partners. In the 2 control groups, a subject also plays with four subjects, but he/she randomly encounters his/her neighbours in each round. At each round, a subject submits his/her choices for *p* and *q* simultaneously (0 ≤ *p*, *q* ≤ 100), and plays the standard UG with each of his/her neighbours with the two different roles. Subjects’ choices are applied to all their neighbours in a round, i.e., a subject makes the same offer and acceptance level to all of his/her neighbours. Thus, our experimental setting is same as many previous theoretical models^19,21–25^.

**Figure 1:**
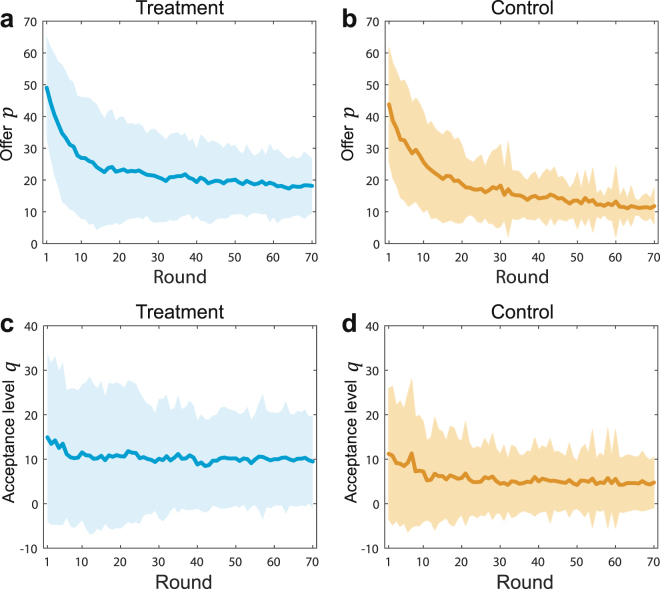
Time evolution of mean values and standard deviations of offers *p* and acceptance levels *q*. (**a**,**b**) Mean values and standard deviations of offers *p* from round 1 to round 70. Fair splits emerge at the beginning of all groups. The mean values of offer *p* decrease rapidly as the game progresses. The mean values in the treatment groups are larger than the control groups. The result indicates that fixed interaction structures can enhance the fairness compared with well-mixed populations. (**c**,**d**) Mean values and standard deviations of acceptance levels *q* from round 1 to round 70. Mean values of acceptance levels *q* are quite low from beginning to end. The mean values of *q* are stable in the treatment groups while the mean values of *q* slight decrease in the control groups. The results demonstrate that most of responders are quasi-rational.

We begin by analyzing subjects’ offer *p* and acceptance level *q*. The time evolution, spatio-temporal patterns and distributions of *p* and *q* are shown in Fig. 1 and Supplementary Figures 4, 5, respectively, and histograms of *p* and *q* at rounds 1, 35 and 70 are shown in Supplementary Figure 6. These figures show that the mean values of *p* decrease over rounds. To be specific, in both the treatment and control groups, the mean value of *p* is about 50 at the beginning and it decreases faster in the control groups than the treatment groups (see power law regressions in Supplementary Figure 7). In contrast, the mean value of *q* keeps constant at about 10 over all the 70 rounds in the treatment groups, and decreases from 10 to 5 in the control groups. The mean values of *p* and *q* in the treatment groups are significantly higher than those of the control groups (see Table 1). These results imply that fixed interaction structures have some effects on promoting fairness as compared to well-mixed populations, although *p* and *q* in these dual-role experiments are significantly lower than the values found in single-role experiments and in most theoretical models (see a comparison in Supplementary Table 2). Furthermore, the standard deviations of *q* over all rounds and over the last 35 rounds are similar in both treatment groups (F-test, *P*-value = 0.5929) and control groups (F-test, *P*-value = 0.3576), which implies that the diversity of *q* does not change significantly along rounds. The standard deviation of *p* over all rounds and over the last 35 rounds in the treatment groups have no significant difference (F-test, *P*-value = 0.2023), however, in the control groups the standard deviation of *p* over last 35 rounds is significantly lower than that over all rounds (F-test, *P*-value < 0.001) (see Table 1). This is consistent with the single-role UG study^31^ that a fixed interaction structure promotes the diversity of offers *p* but not of acceptance levels *q*.

**Table 1:** The mean values and standard deviations of offers and acceptance levels.

	1-70 rounds/1-35 rounds/36-70 rounds	
	Treatment	Control
Mean(*p*)	22.78/26.37/19.18	17.78/22.52/13.03
Mean(*q*)	10.46/10.99/9.92	5.70/6/50/4.49
SD(*p*)	9.23/11.92/8.45	5.40/8.65/3.26
SD(*q*)	8.19/10.05/8.50	5.82/7.35/5.32

We calculate mean values and standard deviations of *p* and *q* for all 70 rounds, and separately for rounds 1 to 35 and rounds 36 to 70. Mean(*p*) and SD(*p*) represent the mean value and the standard deviation of offers of all proposers, respectively, in which a proposer’s offer *p* is taken as the average of his/her offers *p* over 1-70 rounds/1-35 rounds/36-70 rounds. Similarly, Mean(*q*) and SD(*q*) represent the mean value and the standard deviation of acceptance levels *q* of all responders, respectively, in which a responder’s acceptance level is taken as the average of his/her acceptance levels *q* over 1-70 rounds/1-35 rounds/36-70 rounds.

Furthermore, we find there are positive relationships between mean payoffs and rounds in both treatment groups and control groups, and the average values of payoffs over all rounds are 90.47 and 92.14 in the treatment groups and control groups, respectively. Moreover, there are negative relationship between standard deviations of payoffs and rounds in the treatment groups and control groups (see Supplementary Figure 8). The results of payoffs indicate that coordinating behaviours shown in our experiments can enhance cooperative bonus and reduce payoff differences which lines up with some earlier observations^32,33^. In fact, one could see our setup as a related one to the consensus experiments in^33^, where players are incentivized to choose the same colour as their neighbours. However, the fact that there are very many available options to the participants in our setup make it difficult to carry the analogy beyond a general resemblance.

Going from the aggregate to the individual level, let us now look at the behavioural patterns of proposers and responders. In our dual-role UG experiments, the proportion of rational behaviours of responders (i.e., do not reject *q* = 0 or only reject zero offer *q* = 1) is quite high compared with those of single-role experiments. In both the treatment groups and control groups, about half of the responders are rational in the first round (see Supplementary Figures 9–11). Furthermore, we observe that the proportion of rational responders decays over time in the treatment groups, while in the control groups the proportion remains constant (see Supplementary Figure 11). All in all, the proportion and evolution of rational responders is not very different in the treatment and control groups, and therefore the interaction structure does not seem to have large influence on this aspect.

On the other hand, proposers, who in principle should offer the minimum possible amount, seem to behave in a rational, best-response manner, offering the amount that maximizes their payoff given the acceptance levels of their neighbours in the previous round (see Supplementary Note 1.4 for the definition of best-response behaviour). In all groups, the proportions of rational behaviours among proposers are quite low at the beginning, but these proportions increase significantly over rounds and reach about 50 percent at the end (see Supplementary Figures 10,11). Thus, behaviours of proposers show a clear learning trend. As subjects gain experience from repeated observations, they make more precise estimates of the best response offer.

Distributions of individual strategies in the dual-role UG are shown in Supplementary Figure 5. In both the treatment groups and control groups, strategies around (20, 0) and (10, 0) are most popular. Furthermore, in the treatment groups, about 5% of strategies are close to (20, 20), but in the control groups, the proportion is less than 3%. A typical measurement for individual strategies in the dual-role UG is empathy. An empathic individual will offer an amount that is equal to his/her minimum acceptance level^17,20,23,25^. We use this approach to define empathic behaviours (*p* = *q*) as well as altruistic behaviours (>*q*, i.e., offer more than expected) and selfish behaviours (*p* < *q*, i.e., offer less than expected). It has been shown in theory that empathy can emerge spontaneously if there is a fixed interaction structure and the role of proposer or responder is randomly changed from round to round^25^. In our experiments, although the proportion of empathic behaviours slightly increases with rounds (which results in the decline of *p*), the proportion of altruistic behaviours is much higher than the other two types of behaviours (76.37% in the treatment groups and 83.84% in the control groups, see Supplementary Figure 12). Specifically, the difference between *p* and *q* keeps stable at about 10 in the second half of the games in all the groups (see Fig. 1 and Table 1). Furthermore, selfish behaviours have the lowest single round average payoff in both treatment groups and control groups. The average payoff of empathic behaviours is slightly higher than that of altruistic behaviours in the treatment groups, but there is no significant difference between the payoffs of altruistic behaviours and empathic behaviours in the control groups (see Supplementary Figure 13).

**Figure 2:**
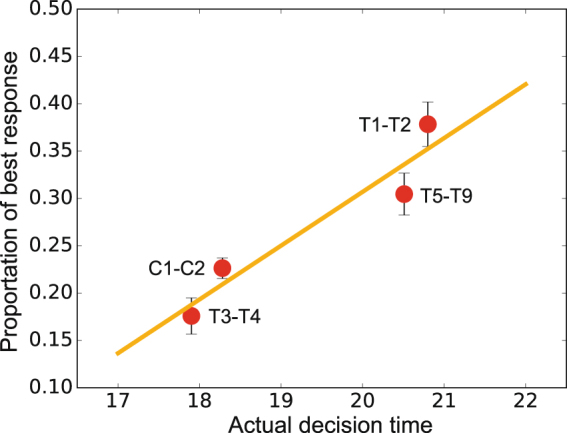
Relationship between best-response behaviours and actual decision time. We analyze the data by classifying the 11 groups into 4 categories depending upon the experimental settings, namely, T1-T2 (large groups with 45 seconds maximum time allowed), T3-T4 (median groups with 30 seconds maximum time allowed), T5-T9 (small groups with 45 seconds maximum time allowed) and C1-C2 (large groups with 45 seconds maximum time allowed). Plotting proportion of best-response behaviours as a function of actual decision time in the 4 categories shows a clear positive correlation. The line is the result of linear regression by using the least squares approach. Error bars denote mean ± s.e.m.

Although the above analysis is informative as to why *p* declines but *q* does not, the reason behind these rational behaviours is still unknown. Previous studies have indicated that individual behaviours in games involving fairness and cooperation are sensitive to decision time, it is then important to investigate the effect of time pressure on decision making^34–37^. Overall, the mean decision time decreases over rounds (see Supplementary Figure 14 and Supplementary Table 3). This means that subjects make decisions faster as they play the game repeatedly. Interestingly, there is a clear positive correlation between actual decision time and proportions of rational behaviours of proposers (Pearson correlation, coefficient =0.9549, *P*-value = 0.0451, see Fig. 2). Note that a subject’s best response choice for *p* (denoted by BR (*p*)) depends on his/her neighbours’ strategies in the previous round. Therefore, it makes sense that shorter decision times decrease the ratio of rational behaviours since BR (*p*) may needs to be recalculated at each round. Since the actual decision time decreases over rounds, we further look at the relationship between relative decision time (i.e., actual decision time minus mean decision time in that round) and the proportion of best-response behaviours (Supplementary Figure 15). In T1-T2 and T5-T9, both faster and slower decisions are more likely to be best-response. The reason is simple. If a subject’s neighbours’ acceptance levels are stable, then his/her optimal offer does not change via rounds so he/she can make a fast decision. In contrast, if the acceptance levels are unstable, then the subject needs more time to calculate the optimal offer so he/she will make a slow decision. Supplementary Figure 14 further reveals that subjects in T3-T4 do not have enough time to make a slow decision. This explains why the proportion of best-response behaviours in T3-T4 is lower than T1-T2 and T5-T9. Moreover, when proposers do not best respond, their offers are often higher than the best-response (i.e., best-response in general leads to smaller *p*). We find a weak negative correlation between the discrepancy *p* − BR(*p*) and decision time (Pearson correlation, coefficient = −0.1412, *P*-value = 0.0117). This result agrees well with the observation in Cappelletti *et al*.^35^, where proposers are likely to make higher offers under time pressure. In contrast, because a subject’s optimal *q* does not change via rounds (always *q* = 0 or 1), the proportions of rational behaviours of responders are affected little by the decision time.

We now analyze the effects of population size on individual behaviors. Supplementary Tables 4, 5 show that the mean values of *q* in different treatment groups are similar. In contrast, the mean value of *p* in T3-T4 is higher than T1-T2 and T5-T9, but the difference between T1-T2 and T5-T9 is not significant. Moreover, we find there is no significant difference in proportion of best-response behaviours between T1-T2 and T5-T9 (see Supplementary Table 6). Overall, subjects in T1-T2 and T5-T9 display very similar behaviours. This implies that the population size does not affect the pattern of individual behaviours. In addition, *p* in T3-T4 is higher because the average decision time in T3-T4 is shorter, which affects the proportion of best-response behaviours.

**Figure 3:**
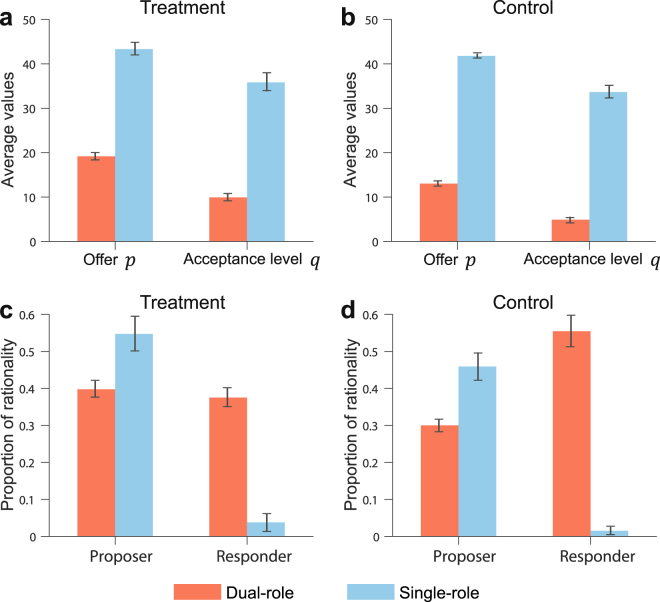
Comparison between dual-role UG experiments and single-role UG experiments. We analyze the data of the last 35 rounds in the treatment and control groups of the dual-role UG experiments and the last 30 rounds in the treatment and control groups of the single-role UG experiments. For each role, there are 318 samples in the dual-role UG experiments and 100 samples in the single-role UG experiments. (**a**) Mean values of *p* and *q* in the dual-role UG treatment groups and single-role UG treatment groups which include 50 proposers and 50 responders. (**b**) Mean values of *p* and *q* in the dual-role UG control groups and single-role UG control groups which include 50 proposers and 50 responders. (**c**) Proportions of rational behaviours of proposers and responders in the dual-role UG treatment groups and single-role UG treatment groups. (**d**) Proportions of rational behaviours of proposers and responders in the dual-role UG control groups and single-role UG control groups. Error bars indicate ±1 s.e.m.

Finally, it is interesting to compare the results of the dual-role UG experiments with our previous single-role UG experiments^31^ (two treatment groups with fixed interaction structures and two control groups with well-mixed populations, total *n* = 200, including 100 proposers and 100 responders, 60 rounds, see Supplementary Note 1.5 for the design of the single-role UG experiments). We use the data of the last 35 rounds in the control and treatment groups of the dual-role UG experiments and the last 30 rounds in the control and treatment groups of the single-role UG experiments. We first compare *p* and *q* between the dual-role UG experiments treatment groups and the single-role UG experiments treatment groups. As shown in Fig. 3a, both *p* and *q* in the dual-role UG experiments treatment groups are much lower than in the single-role UG ones (*p*: 19.18 vs 43.33, Mann-Whitney U-test, *P*-value < 0.001; *q*: 9.92 vs 35.83, Mann-Whitney U-test, *P*-value < 0.001). The same is true for well-mixed populations, dual-role UG experiments control groups compared to single-role UG experiments control groups (see Fig. 3b, *p*: 13.03 vs 41.80, Mann-Whitney U-test, *P*-value < 0.001; *q*: 4.90 vs 33.65, Mann-Whitney U-test, *P*-value < 0.001). These observations support our first general conclusion that equal status in the UG is actually detrimental in terms of fairness. This is likely to arise from the fact that single-role UG responders can only increase their payoff by rising their acceptance level, thus forcing proposers to increase their offers; on the contrary, when playing both roles, subjects would mainly focus on the offer in order to increase their payoffs, instead of trying to do it by rising their acceptance levels. This is supported by what we observe in the first round of our dual-role experiments, i.e., proposers choose fair offers but responders agree to accept low offers. In particular, this observation is consistent with those by Oxoby and McLeish^29^, where they found that people are more likely to accept low offers in a strategy method UG compared to a sequential decision UG.

In agreement with the explanation above, Fig. 3c shows that the proportion of rational behaviours of responders in the dual-role UG experiments treatment groups is much higher than the proportion in the single-role UG experiments treatment groups (37.53% vs 3.80%). However, the proportion of rational behaviours of proposers (best-response behaviours) in the dual-role UG experiments treatment groups is lower than the proportion in the single-role UG experiments treatment groups (39.78% vs 54.73%, Mann-Whitney U-test, *P*-value = 0.0027). For well-mixed populations, we find similar but even more dramatic results (see Fig. 3d): The proportion of rational behaviours of responders in the dual-role UG experiments control groups is much higher than the proportion in the single-role UG experiments control groups (55.47% vs 1.53%), but the proportion of rational behaviours of proposers (best-response behaviours) in the dual-role UG experiments control groups is lower than in the single-role UG experiments control groups (30.03% vs 45.93%, Mann-Whitney U-test, *P*-value < 0.001). This indicates that the acceptance level choices in the populations may be driving the offers, and that single-role UG responders move away from rationality in an attempt to increase their payoffs.

## Discussion

In sum, our experiments show that equal status promotes rational splits, and that a fixed interaction structure has positive effects on fairness. Responders keep their acceptance level roughly constant and low all along the experiment, and proposers grow more inclined towards a best-response approach as the experiment proceeds. The best-response behaviour leads to fairer offers in the structured population than in the well-mixed population, although the values are far from what most theoretical models predict^19,21–25^ (see a comparison in Supplementary Table 2).

We note that the best-response offer for a proposer must be equal to one of his/her neighbors’ acceptance levels, and in many cases, it coincides exactly with the maximum acceptance level. We then calculate the proportion of behaviours that offer the maximum acceptance level, finding that this proportion is slightly lower than that of the best-response behaviours (see Supplementary Notes 1.6). This reveals that some subjects indeed choose their offers based on the best-response consideration rather than simply adopt their neighbors’ maximum acceptance level. In addition to a large amount of best-response behaviours, there are also many subjects that choose a *p* value that is 1–5 higher than the best-response offer (see Supplementary Figure 9). This type of behaviour can be explained by a reinforcement learning model, where proposers update their offers based on trial-and-error and responders randomly pick their acceptance level from a database which is built upon the experimental data (see Supplementary Notes 1.7). The simulation results match the experimental results quite well. In both the structured and well-mixed populations, *p* declines fast in the first 20 rounds, but remains constant or decreases slowly in the last 20 rounds (see Supplementary Figure 16). This is consistent with previous studies showing that reinforcement learning can explain human behaviours in the spatial prisoner’s dilemma experiments^38,39^.

Models based on evolutionary game theory fail to predict the experimental results because most subjects in the experiments used best-response like strategies or trial-and-error rather than imitation. Rejecting unfair offers can reduce the payoff difference between a subject and his/her neighbors but cannot improve his/her own payoff. Therefore, higher *q* can spread through (local) imitation, but subjects based on payoff considerations prefer to choose lower *q*. In particular, the proportions of rational behaviours of responders decrease over time in the treatment groups but remain constant in the control groups (see Supplementary Figure 11). This may be because some rational responders in the treatment groups raise their *q* to fit the minimum offer of their neighbors (see Supplementary Figure 6). Note that such change is riskless when their neighbors’ offers are stable. In contrast, rational responders keep low acceptance level all the time in the control groups because they don’t know the history offers of their opponents. This explains why a fixed interaction structure has some effects on promoting fairness.

Interestingly, the result of the dual-role UG is very different from the single-role UG experiment^31^ where, when subjects play only as responders, they depart considerably from rationality, in contrast with what we observe here. This in turn raises an interesting question. In the single-role UG, the statuses of subjects are unequal, but they still seem to acknowledge that the responders should obtain about 40% of the pot, which is close to the fair split. By contrast, although subjects have equal status in the dual-role UG, subjects forgo getting large payoffs as responders. It thus seems that when there is no fundamental inequity in initial allocation, offers are more salient to subjects. This is probably because they are perceived as an ‘active’ choice, while the acceptance level may be regarded as more ‘passive’ and then less useful to improve the subjects’ payoffs. In contrast, in the single-role UG, responders’ payoffs depend entirely on proposers making substantial offers. Thus, responders can only increase their payoff by rising their acceptance level. Another possible explanation would be that the unequal status in the single-role UG activates inequity aversion or ‘negative’ emotions of responders^40,41^, while equal status in the dual-role UG promotes rational thinking. Whatever the ultimate reasons behind these anomalous behaviours, the fact that subjects adjust their offers to their environments while keeping their acceptance levels low can provide a sound basis on which new, more accurate theoretical approaches to understand the evolution of fairness can be designed.

## Methods

We conducted a series of repeated dual-role UG experiments, including 9 treatment groups T1-T9 with structured populations and 2 control groups C1-C2 with well-mixed populations, in computer labs at Beijing Normal University. All 321 subjects were freshmen and sophomores recruited from Beijing Normal University that had not taken courses on game theory or economy. The interactions were anonymous, and via computers. In the 9 treatment groups, every subject plays with four fixed partners. To be precise, each participant occupies a location of a static 4-degree ring structure and plays the dual-role UG with his/her four immediate neighbours (see Supplementary Figures 3). In addition, a sketch map of the ring structure is showed to subjects. Thus, subjects knew that any two of them don’t have exactly the same neighbours, but they are not completely independent. In the 2 control groups, a subject also plays with four subjects, but he/she randomly encounters his/her neighbours in each round. At each round, a subject submits his/her choices for *p* and *q* simultaneously (0 ≤ *p*, *q* ≤ 100), and plays the standard UG with each of his/her neighbours with the two different roles. To be specific, when a subject plays proposer, all his/her neighbours play responders, and when he/she plays responder, all his/her neighbours play proposers.

In the dual-role UG, a subject’s total points interacting with one of his/her neighbours are the sum of his/her points obtained as a proposer and as a responder. For instance, denote the strategy of subject *i* by (*p*_*i*_, *q*_*i*_). Then the points of subject *i* interacting with neighbor *j* can be denoted as follows

1 $${U}_{ij}=\{\begin{array}{ll}{p}_{j}+100-{p}_{i} & {p}_{i}\ge {q}_{j}\,and\,{p}_{j}\ge {q}_{i}\\ 100-{p}_{i} & {p}_{i}\ge {q}_{j}\,and\,{p}_{j} < {q}_{i}\\ {p}_{j} & {p}_{i} < {q}_{j}\,and\,{p}_{j}\ge {q}_{i}\\ 0 & {p}_{i} < {q}_{j}\,and\,{p}_{j} < {q}_{i},\end{array}$$

where *p*_*i*_, *p*_*j*_, *q*_*i*_, *q*_*j*_ ∈[0, 100]^15–17^. In the experiments, the payoff of a subject (in a round) is taken as the average points of his/her four interactions. That is, subject *i*’s payoff can be calculated as ${U}_{i}={\sum }_{j\in {{\rm{\Gamma }}}_{{\rm{i}}}}{U}_{ij}/4$, where Γ_*i*_ is the set of his/her neighbours. At the end of each round, subjects are informed of their neighbours’ choices and payoffs (see Supplementary Figures 2 for more details). Note that this differs from setups in which there is competition among responders or proposers, as all deals that verify that the offer is larger than the acceptance level are actually realized.

### Ethics

All participants provided written informed consent. All experimental methods were carried out in accordance with the approved guidelines. All experimental protocols were approved by the Ethics Review Committee of Beijing Normal University.

## Electronic supplementary material

Supplementary Information
