# Supplementary material for: Equal status in Ultimatum Games promotes rational sharing
Source: Sci Rep. 2018 Jan 19;8:1222. doi: 10.1038/s41598-018-19503-x (PMC5775192; doi:10.1038/s41598-018-19503-x)
Supplement: Supplementary file 1 — Supplementary Information [file 41598_2018_19503_MOESM1_ESM.pdf]

Supplementary Information for

Equal status in Ultimatum Games promotes rationality

Xiao Han[1,2], Shinan Cao[3], Jian-Zhang Bao[2], Wen-Xu Wang[2], Boyu Zhang[4], Zi-You Gao[1], and Angel Sánchez[5,6,7]

[1]*MOE Key Laboratory for Urban Transportation Complex Systems Theory and Technology, Beijing Jiaotong University, Beijing, 100044, P. R. China*
[2]*School of Systems Science, Beijing Normal University, Beijing, 100875, P. R. China*
[3]*School of Finance, University of International Business and Economics, Beijing, 100029, P. R. China*
[4]*School of Mathematical Sciences, Beijing Normal University, Beijing, 100875, P. R. China*
[5]*Grupo Interdisciplinar de Sistemas Complejos (GISC), Departamento de Matemáticas, Universidad Carlos III de Madrid, Spain*
[6]*UC3M-BS Institute of Financial Big Data (IFIBID), Universidad Carlos III de Madrid, Spain*
[7]*Instituto de Biocomputación y Física de Sistemas Complejos (BIFI), Universidad de Zaragoza, Spain*

Contents

1 Supplementary Notes **2**
1.1 Experimental settings for dual-role ultimatum game experiments 2
1.2 Experimental instructions for T1-T9 2
1.3 Experimental instructions for C1-C2 3
1.4 Calculating best-response behaviour 4
1.5 Experimental settings for single-role ultimatum game experiments 4
1.6 Calculating ‘offering the maximum acceptance level’ behaviour 5
1.7 Reinforcement learning model 5

2 Supplementary Figures **7**

3 Supplementary Tables **21**

4 Supplementary References **24**

1 Supplementary Notes

1.1 Experimental settings for dual-role ultimatum game experiments

We conducted a series of repeated dual-role UG experiments, including 9 treatment groups with structured populations and 2 control groups with well-mixed populations in computer labs of Beijing Normal University. Detail settings of the groups are shown in Supplementary Table 2. All 321 subjects were freshmen and sophomores recruited from Beijing Normal University without taking classes of game theory and economy. The interactions were anonymous, and via computers. Frosted glass dividers ensured that the students could not see each other (see Supplementary Figure 1). We built the experimental platform by using PHP, mySQL and javascript, and ran the platform programs on the server. Schematic diagrams of our experimental platform are shown in Supplementary Figure 2.

Before starting the experiment, we explained the game to all subjects, including the rules of the game, the purpose of the game, and the feedback information in the computer in 20 minutes. All subjects in each session were given the same instructions (in Chinese). To ensure that all subjects fully understand the game, we implemented 2 exercises and 5 practice rounds before the formal experiment (last about 10 minutes). During the period, all subjects can raise their hands, and our experimenters would answer their questions. The formal experiment lasted about 60 minutes, and subjects were not told the number of rounds, so as to avoid end round effects. Each round is time limited (except for the first round). In T3-T4, subjects have 30 seconds to submit their decisions at each round, and in other groups, subjects have 45 seconds to submit their decisions. Subjects knew that if they did not decide within the given time, they would be allocated their own decisions in the previous round. Since the subjects had familiarized themselves with the game during the practice rounds, this happened only 412 times in 24672 decisions (1.67 %). After the experiment, the total payoffs of each subject obtained in the formal experiment was converted to Chinese Yuan at a ratio of 100 : 1. This pay plus 30 Chinese Yuan is his/her final income (see Supplementary Table 2 for details).

In all experiments, the data of three subjects (two in C1 and one in T3) are excluded because we notice that they do not really understand the game. To keep the comparison unbiased, all results were calculated using data in 1-70 rounds.

1.2 Experimental instructions for T1-T9

Welcome and thanks for participating in this game. Please read the game instruction carefully. If you have any questions please raise your hand. One experimenters will then come to you and answer your questions. From now on, communication with other participants is not allowed. Please switch off your mobile phone and keep quiet in the whole game.

You will play a decision making game. In the game, you would not know other persons' true identity. Your scores depend on your and your partners' decisions. Your final income = fixed income 30 Chinese Yuan + 0.01 × total scores.

Game instruction

1. In this game, you play two roles, proposer and responder, and submit your offer and demand simultaneously. You play the game with four fixed partners, who also play the two roles.
2. At each round, you play the game twice with different roles at the same time. When you play proposer, all your partners play responders; when you play responder, all your partners play proposers.
3. A proposer and a responder share 100 points. If proposer' offer greater than or equal to responder' demand, the proposer receives (100−**proposer's offer**), and the responder receives **(proposer's offer)**, otherwise, both receive 0.
4. Your total points are the sum of your four interactions. Your score = (your total points)/(number of partners). After all the participants submit their offer and demand, the system will calculate your points obtained as a proposer, your points obtained as a responder, your total points, and your scores.

Example (A sketch map of the ring structure is showed to subjects.)

1. Suppose you have four partners, A, B, C and D.
2. At each round, you play the game with all your four partners.
3. You submit your offer p and demand q.
4. Suppose the four partners' offers are p_A, p_B, p_C and p_D, and demands are q_A, q_B, q_C, q_D.
5. Suppose your offer p satisfies $q_C, q_D > p \geq q_A, q_B$. Then, as a proposer, your offer is accepted by A and B, and your points obtained as a proposer are $(100 - p) + (100 - p)$.
6. Suppose your demand q satisfies $p_A < q \leq p_B, p_C, p_D$. Then, as a responder, you accept offers from B, C and D, and your points obtained as a responder are $p_B + p_C + p_D$.
7. In this round, your total points are $(200 - 2p) + p_B + p_C + p_D$.
8. Your scores are $[(200 - 2p) + p_B + p_C + p_D]/4$, where 4 is the number of your partners.

Exercise 1

Now we generate your and your partners' offers and demands randomly. For simplicity, we only generate multiples of 10 for offers and demands. You need calculate your points obtained as a proposer, your points obtained as a responder, your total points and your scores. Different subjects may have different partners, so you cannot calculate your partners' scores.

Exercise 2

Same as Exercise 1.

1.3 Experimental instructions for C1-C2

Game instruction

1. In this game, you play two roles, proposer and responder, and submit your offer and demand simultaneously. You play the game with four partners, who also play the two roles.
2. At each round, you play the game twice with different roles at the same time. When you play proposer,

all your partners play responders; when you play responder, all your partners play proposers.
3. A proposer and a responder share 100 points. If proposer' offer greater than or equal to responder' demand, the proposer receives (100−**proposer's offer**), and the responder receives **(proposer's offer)**, otherwise, both receive 0.
4. Your total points are the sum of your four interactions. Your score = (your total points)/(number of partners). After all the participants submit their offer and demand, the system will calculate your points obtained as a proposer, your points obtained as a responder, your total points, and your scores.
5. At the beginning of each round, you will randomly encounter four new partners.

[The rest parts are same as *Instructions for T1-T2*.]

1.4 Calculating best-response behaviour

We used a rigorous definition of best-response behaviour to identify whether proposers were rational. The best strategy for rational proposers in each round was to offer the amount that maximizes payoff, keeping in mind the acceptance levels of indicated by neighbouring responders in the previous round [1, 2]. For a proposer with k neighbouring responders whose acceptance levels in the previous round were respectively $q_1, ..., q_k$ (with $q_1 < \cdots < q_k$), the best strategy was $p = \text{argmax}_{q_i}\{i \times (100 - q_i)/k\}$, where $i \times (100 - q_i)/k$ was the payoff if the proposers offered q_i. We found that the proportion of rational proposers gradually increased and eventually nearly about half of all proposers take best-response behaviours in all groups. Our definition of best-response behaviours of proposers was extremely rigorous which indicate that the proportion of rational proposers was quite high in the last several rounds.

1.5 Experimental settings for single-role ultimatum game experiments

In our previous single-role UG experiments, we totally conducted 2 treatment groups and 2 control groups [3]. In each group, we recruited 50 subjects, half of whom were randomly assigned proposers and the rest randomly assigned responders. The interactions were executed via computer and were anonymous. We built the experimental platform by using z-Tree [4]. In the single-role UG experiments, each subject only enacted one role, proposer or responder, and his/her role didn't change during the experiment. In the treatment groups, each subject was assigned in a location within a static bipartite network, and played UG with their neighbours who enacted the other role. All of the proposers' neighbours are responders and vice versa. We use two static bipartite networks including a regular bipartite network in which each node has four neighbors and a random bipartite network in which the number of neighbors ranges from 2 to 6 (with an average degree of 4). In the control groups, the population structure changes and players randomly encounter their neighbours in each round.

In the single-role UG, all subjects must use one decision behavior as they interact with their neighbors; that is, a proposer must make the same offer $p(0 \leq p \leq 100)$ to all of his or her neighboring responders, and a responder must indicate the same minimum acceptance level $q(0 \leq q \leq 100)$ to all of

his or her neighboring proposers. The payoff of a subject (proposer or responder) is taken as the average points of his/her all interactions. That is, a subject i's payoff can be calculated as $U_i = \sum_{j \in \Gamma_i} U_{ij}/k_i$, where Γ_i is the set of his/her neighbours, k_i is the number of his/her neighbours, and U_{ij} is the points of subject i interacting with neighbor j.

1.6 Calculating 'offering the maximum acceptance level' behaviour

We note that the best-response offer for a proposer must be equal to one of his/her neighbors' acceptance levels. In particular, the best-response offer is exactly the maximum acceptance level if $\max\{q_i(t)\} \leq 25$. We then calculate the proportion of behaviours that offer the maximum acceptance level, i.e., $p(t+1) = \max\{q_i(t)\}$. The proportions of 'offering the maximum acceptance level' behaviours are 0.3043 and 0.2250 for the treatment groups and the control groups, respectively. Note that the proportions of best-response behaviours are 0.3047 and 0.2264 for the treatment groups and the control groups, respectively, i.e., the proportion of behaviours that offer the maximum acceptance level is slightly lower than that of the best-response behaviours. This reveals that some subjects indeed choose their offers based on the best-response consideration rather than simply adopt their neighbors' maximum acceptance level.

1.7 Reinforcement learning model

In order to get a deeper insight into this theoretical significance of our experimental results, we have run simulations based on a type of reinforcement learning model [5]. Firstly, we build two databases of all responder acceptance levels obtained from all treatment groups and all control groups, respectively. Then we randomly pick responder acceptance level sequences from the two databases and use reinforcement learning model to reproduce proposers' offer p for treatment groups and control groups, respectively. We use a static 4-degree ring structure and well-mixed population with 50 subjects in treatment simulations and control simulations, respectively. The simulation process of reinforcement learning model is as follows.

1. *Initial propensities*: We reduce the offer set of proposers into $\{0, 5, 10, ..., 100\}$ in our simulations and assume that all proposers have the same initial propensities for all offers p in the simplified strategy set, which are set equal to fair split 50.

2. *Update propensities* : Suppose a proposer i has chosen offer p_k in round t, the propensity in round $t+1$ is updated by

$$Q_i^k(t+1) = \theta u_i^k(t) + (1-\theta)Q_i^k(t), \tag{1}$$

where $Q_i^k(t+1)$ and $Q_i^k(t)$ denote the propensities of proposer i chosen offer p_k at round $t+1$ and round t, respectively, $u_i^k(t)$ is the payoff of proposer i chosen offer p_k at round t, θ is the learning rate which is set to 0.2 in our simulations.

3. *Update probabilities*: The probability of choosing offer p_k in round $t+1$ is determined by

$$P_i^k(t+1) = \frac{e^{\lambda Q_i^k(t+1)}}{\sum_{k=1}^{21} e^{\lambda Q_i^k(t+1)}}, \tag{2}$$

where $k = 1, 2, \cdots, 21$ and λ is a parameter that determines reinforcement sensitivity which is set to 0.2 in our simulations. We repeat step 2 and step 3 until the simulation reaches a predetermined round.

2 Supplementary Figures

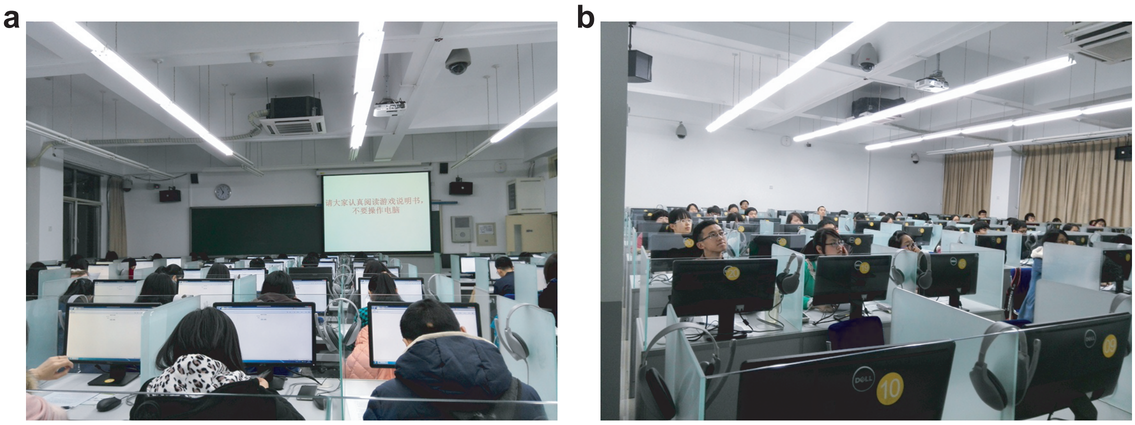

Supplementary Figure 1: Photos of the computer lab. Frosted glass dividers are used to avoid subjects glancing others screens. All subjects can play the game in a quiet environment.

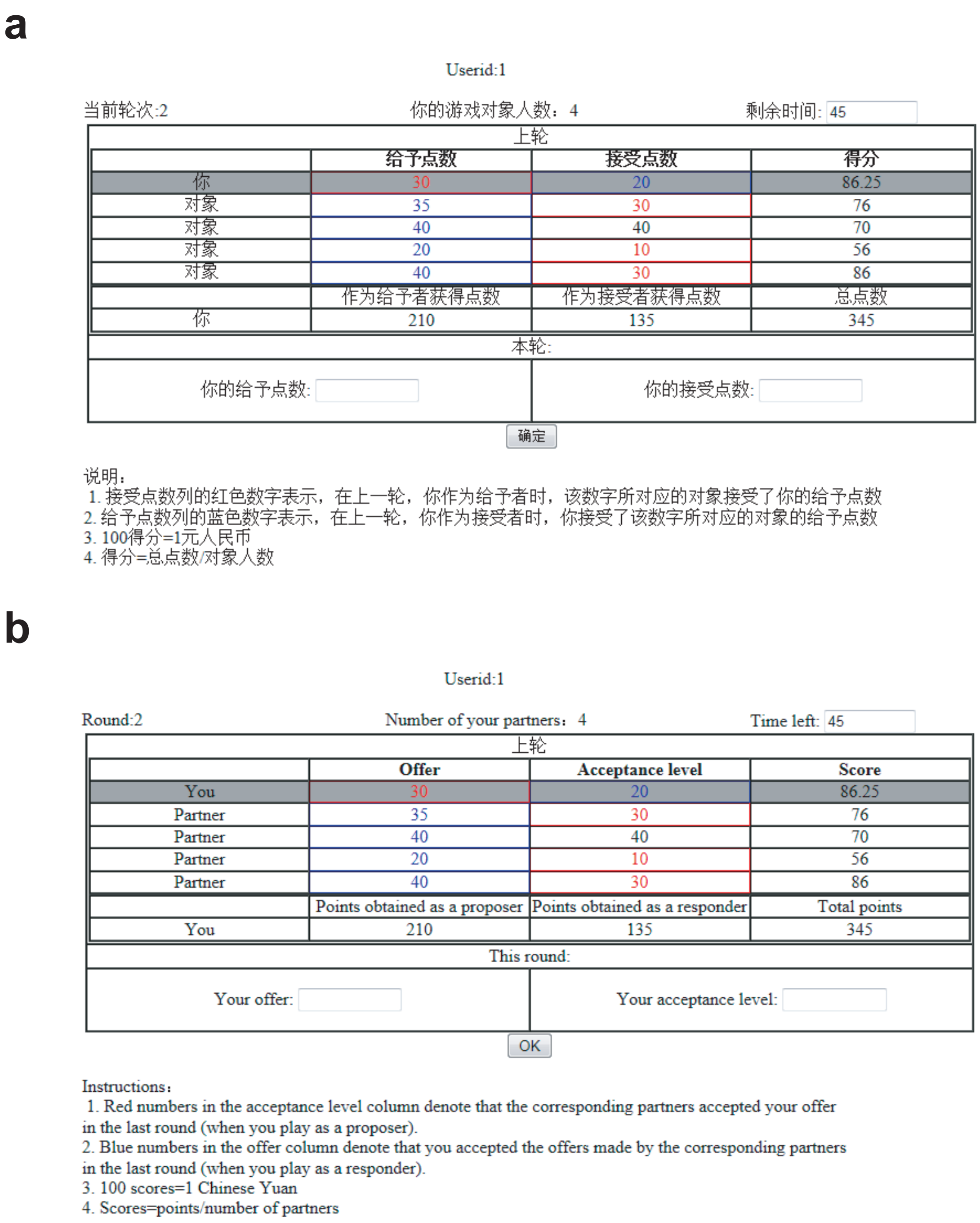

Supplementary Figure 2: Screenshots of the experimental platform. **a**, Chinese version. **b**, English version, translated from the Chinese version. We used the interface in Chinese in the experiments.

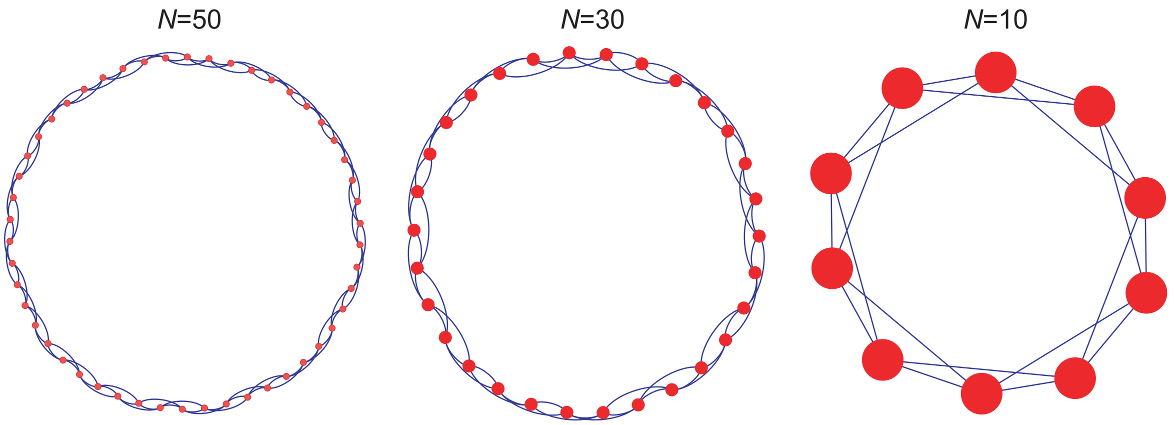

Supplementary Figure 3: Illustrations of the ring structures in the treatments. In the treatment groups, subjects played dual-role UG on the 4-degree ring structures (these figures were showed to subjects). These ring structures have 50 nodes (treatment groups T1-T2, see left), 30 nodes(treatment groups T3-T4, see middle), or 10 nodes (treatment groups T5-T9, see right). We note that two neighbouring subjects have and only have two joint neighbours. Thus, any two subjects don't have exactly the same neighbours, but on the other hand they are not completely independent either.

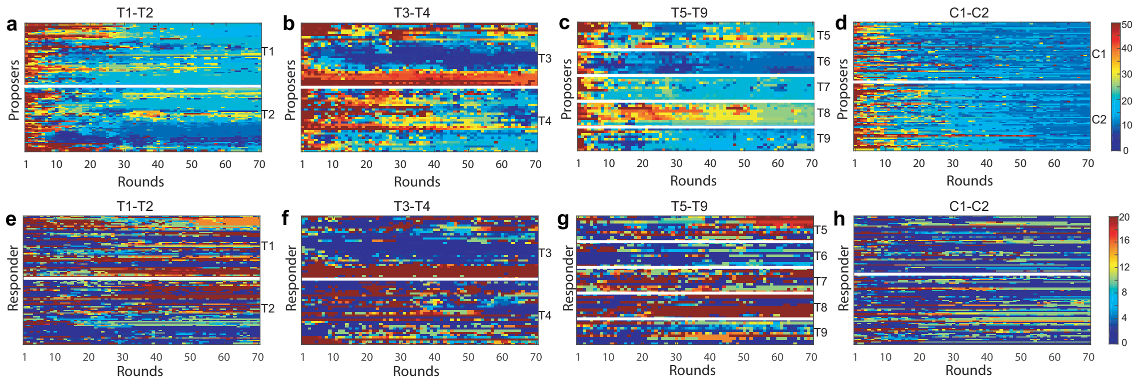

Supplementary Figure 4: Spatio-temporal patterns of proposers' offers and responders' acceptance levels. a-d, Spatio-temporal patterns of the proposers' offer p in T1-T2 (a), T3-T4 (b), T5-T9 (c) and C1-C2 (d). The ordinate represents the spatial orders of proposers. Two proposers with most common neighbours will be adjacent to each other. The color bar represents the value of offer p. **e-f**, Spatio-temporal patterns of the responders' acceptance levels q in T1-T2 (e), T3-T4 (f), T5-T9 (g) and C1-C2 (h). The ordinate represents the spatial orders of responders. Two responders with most common neighbours will be adjacent to each other. The color bar represents the value of acceptance level q.

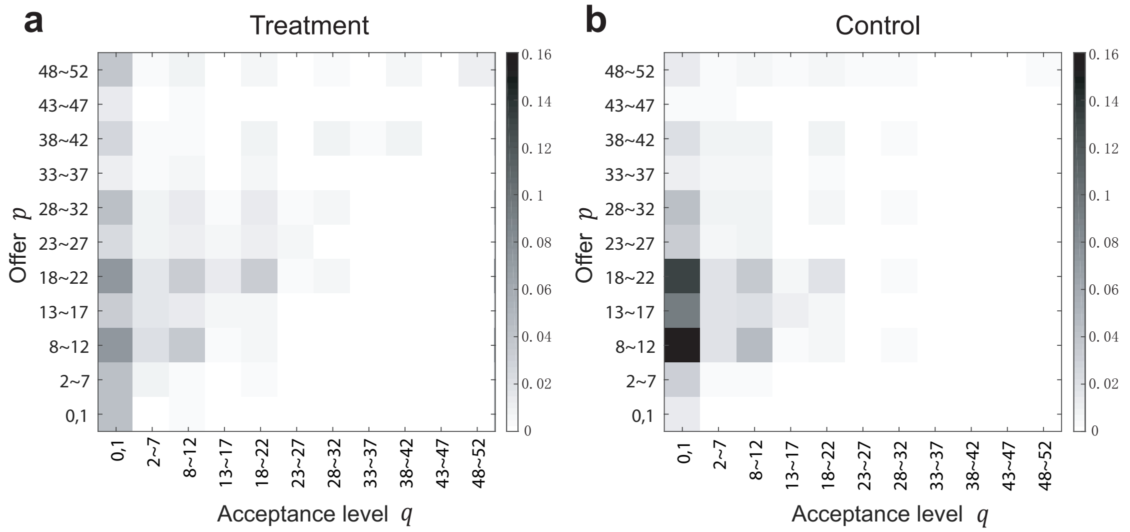

Supplementary Figure 5: Distributions of individual strategies in the dual-role UG. a, The treatment groups, **b**, the control groups.

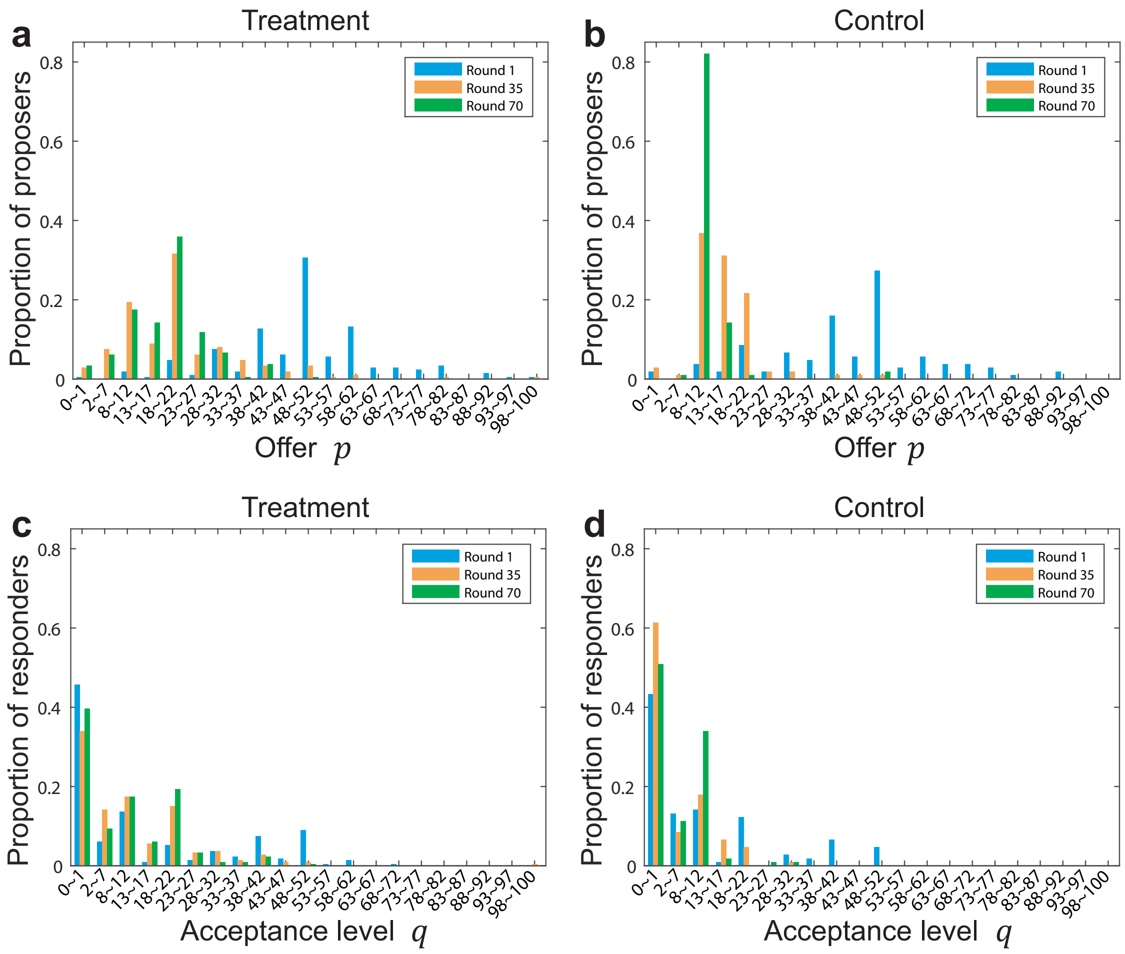

Supplementary Figure 6: Distribution of subjects offers p and acceptance levels q. a-b, Histograms of the proposers' offers p at round 1, round 35 and round 70 in the treatment and control groups, respectively. **c-d**, Histograms of the responders' acceptance levels q at round 1, round 35 and round 70 in the treatment and control groups, respectively.

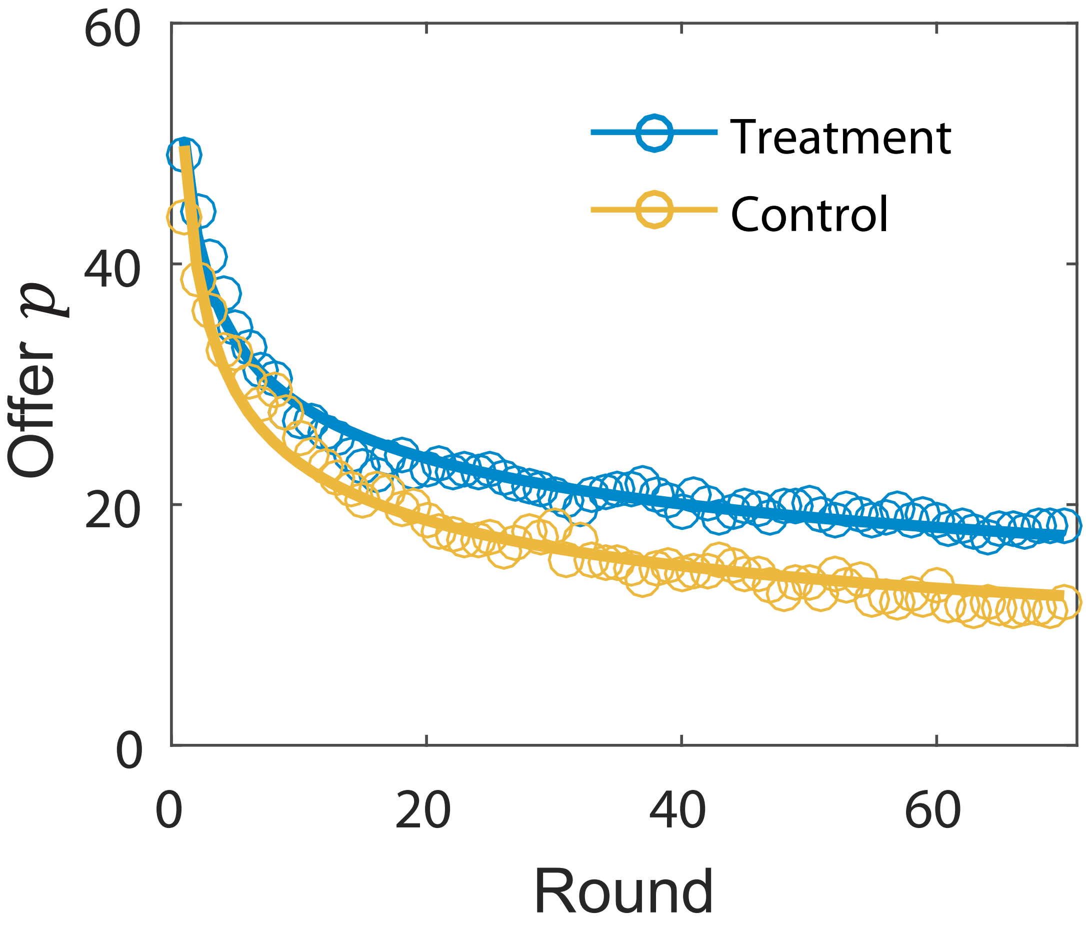

Supplementary Figure 7: Power law regressions for the time evolution of mean values of offers p. In the control groups, $p(t) = 49.81t^{-0.327}$ (t is the round number) with coefficient of determination $R^2 = 0.9576$. In the treatment groups, $p(t) = 50.41t^{-0.250}$ with coefficient of determination $R^2 = 0.9760$. The regression result shows clearly that the mean value of p decreases faster in the control groups than the treatment groups.

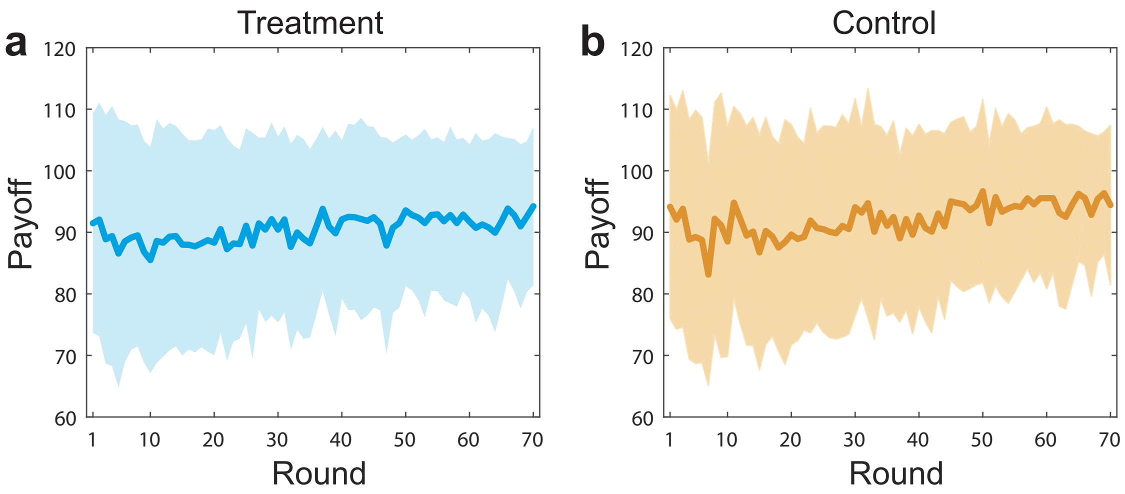

Supplementary Figure 8: Time evolution of mean values and standard deviations of payoffs. (a)Treatment groups, (b) Control groups. The mean payoffs of the treatment groups and control groups are 90.47 and 92.14, respectively. There are positive correlation between the payoffs and rounds in the treatment groups (Pearson correlation coefficient= 0.6657, P-value< 0.001) and control groups (Pearson correlation coefficient= 0.6619, P-value< 0.001). Moreover, the standard deviations of payoffs and rounds have negative correlations in the treatment groups (Pearson correlation coefficient= -0.8514, P-value< 0.001) and control groups (Pearson correlation coefficient= -0.8633, P-value< 0.001).

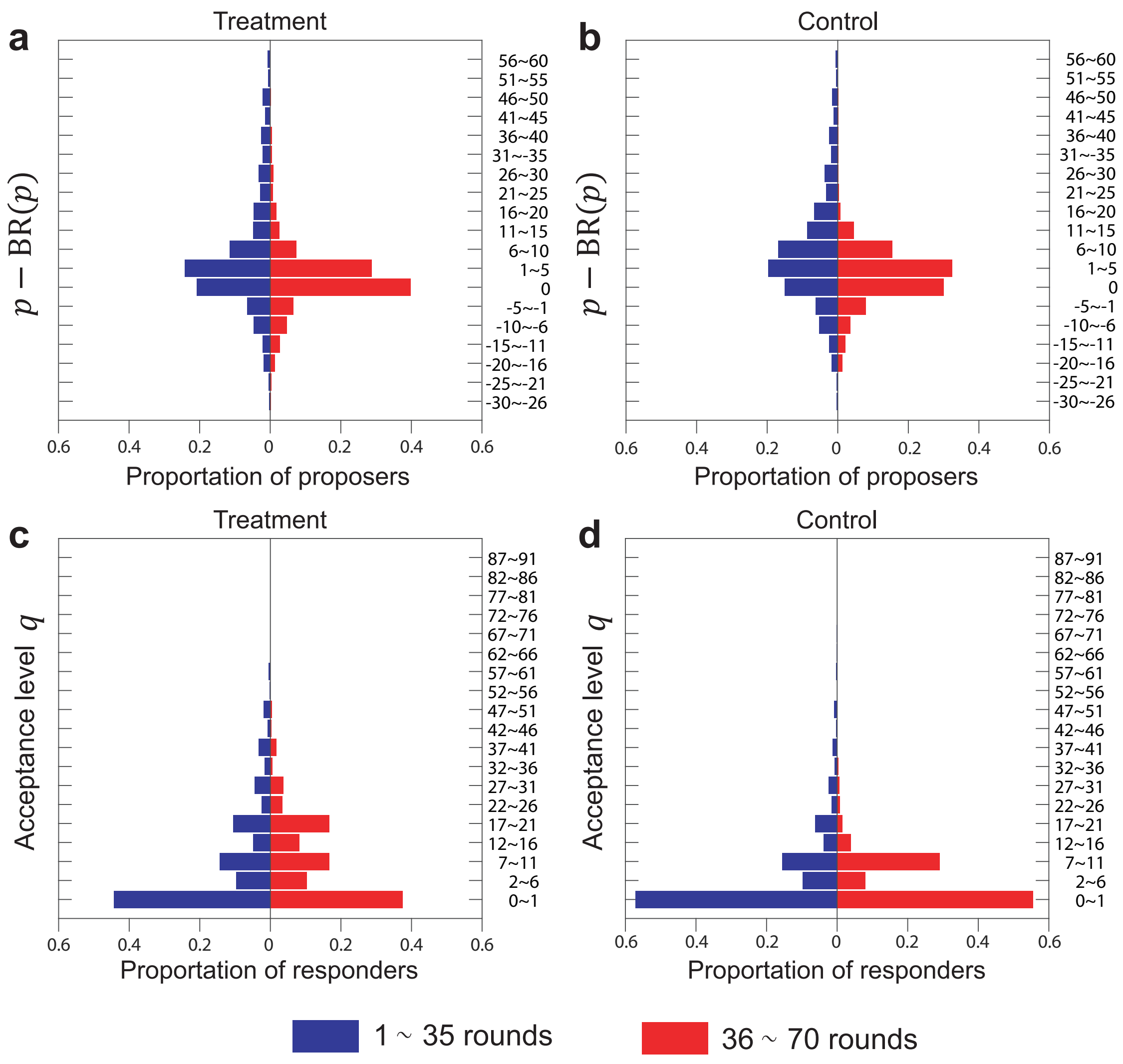

Supplementary Figure 9: Distributions of $p - \mathrm{BR}(p)$ and acceptance level q. We show the distributions of $p - \mathrm{BR}(p)$ and q separately for rounds 1 to 35 and rounds 36 to 70. **a-b**, The distributions of $p - \mathrm{BR}(p)$ in the treatment and control groups, respectively. In all the groups, the proportions of best-response behaviours in the last 35 rounds are higher than that in the previous 35 rounds. **c-d**, The distributions of q in the treatment and control groups, respectively. In all the groups, the distributions in the previous 35 rounds and the last 35 rounds are similar. The result is consistent with Supplementary Figure 7.

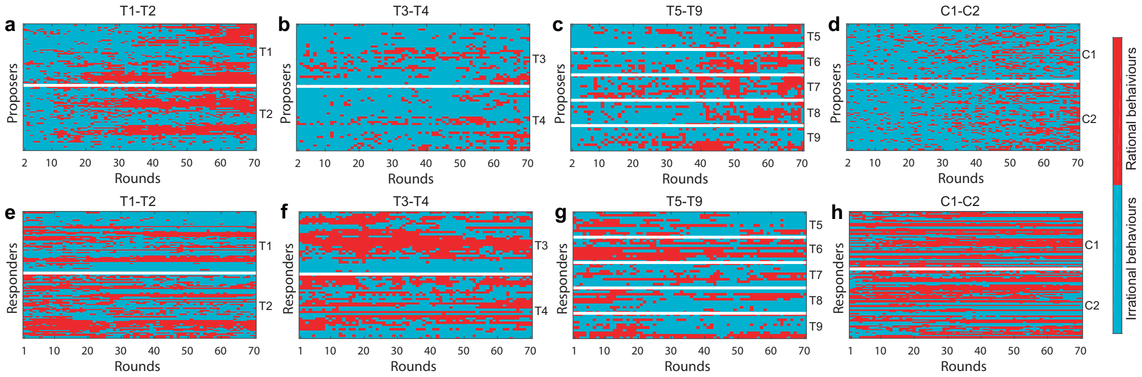

Supplementary Figure 10: Spatio-temporal patterns of rational and irrational behaviours of proposers and responders. **a-d**, Spatio-temporal patterns of rational behaviours (rigorous best-response behaviours) and irrational behaviours of proposers in T1-T2 (a), T3-T4 (b), T5-T9 (c) and C1-C2 (d). The ordinate represents the spatial orders of proposers. Two proposers with most common neighbours will be adjacent to each other. **e-h**, Spatio-temporal patterns of rational behaviours ($q = 0$ or 1) and irrational behaviours of responders in T1-T2 (e), T3-T4 (f), T5-T9 (g) and C1-C2 (h). The ordinate represents the spatial orders of responders. Two responders with most common neighbours will be adjacent to each other. The red color represents rational behaviours and the blue color represents irrational behaviours.

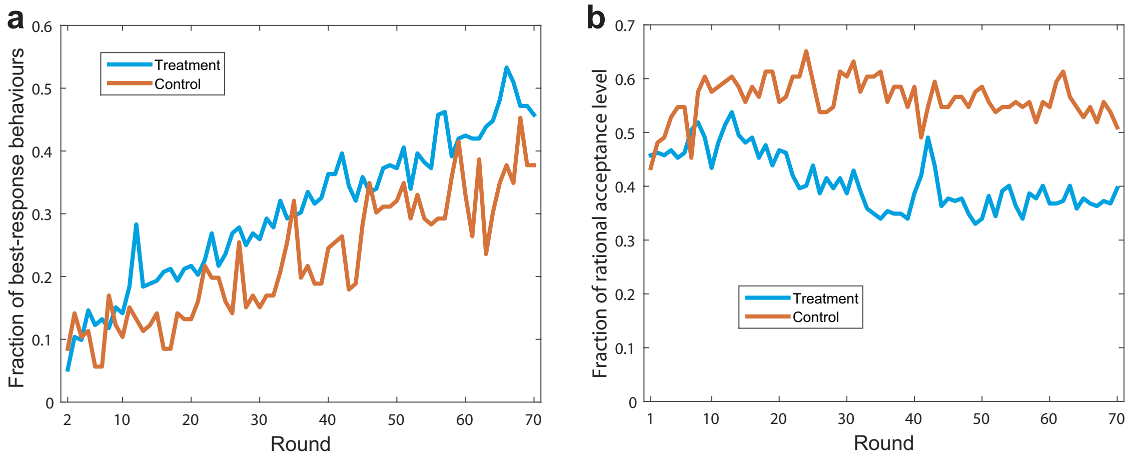

Supplementary Figure 11: Time evolution of fractions of rational behaviours. **a**, The mean values of fractions of rational behaviours of proposers (i.e., best response) are 0.3047 and 0.2264 in the treatment groups and control groups, respectively. The Pearson correlation coefficient between proportions of rational behaviours of proposers are 0.9699 and 0.9011 in the treatment groups and control groups, respectively. **b**, The mean values of fractions of rational behaviours of responders (i.e., q = 0 or 1) are 0.4095 and 0.5628 in the treatment groups and control groups, respectively. The Pearson correlation coefficient between proportions of rational behaviours of responders are -0.7367 and -0.0346 in the treatment groups and control groups, respectively.

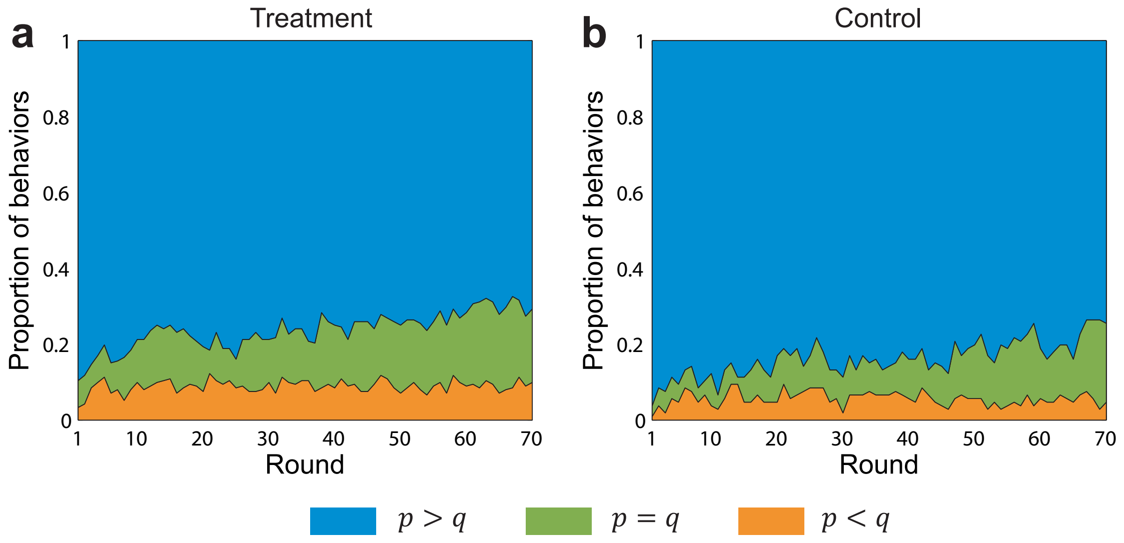

Supplementary Figure 12: Time evolution of the proportions of the three types of behaviours, namely, altruistic behaviours $p > q$, empathic behaviours $p = q$ and selfish behaviours $p < q$. **a**, In the treatment groups, the mean proportion of altruistic behaviours $p > q$, empathic behaviours $p = q$ and selfish behaviours $p < q$ are 76.37%, 14.72% and 8.91%. **b**, In the control groups, the mean proportion of altruistic behaviours $p > q$, empathic behaviours $p = q$ and selfish behaviours $p < q$ are 83.84%,10.55% and 5.61%. Most subjects in our experiments adopt altruistic behaviours.

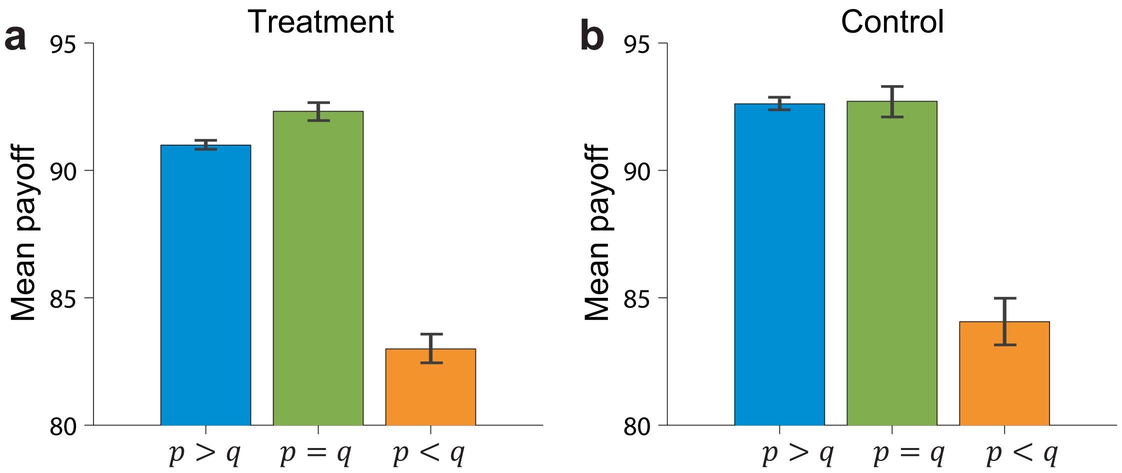

Supplementary Figure 13: Mean payoffs of the three types of behaviours, namely, altruistic behaviours $p > q$, empathic behaviours $p = q$ and selfish behaviours $p < q$. a, In the treatment groups, the mean payoffs of altruistic behaviours $p > q$, empathic behaviours $p = q$ and selfish behaviours $p < q$ are 90.99, 92.31 and 82.99. **b**, In the control groups, the mean payoffs of altruistic behaviours $p > q$, empathic behaviours $p = q$ and selfish behaviours $p < q$ are 92.61, 92.71 and 84.06. The mean payoff of altruistic behaviours $p > q$ is less than that of empathic behaviours $p = q$ in the treatment groups (Mann-Whitney U-test, P-value< 0.001), however, there is no big difference between the mean payoff of altruistic behaviours $p > q$ and empathic behaviours $p = q$ in the control groups (Mann-Whitney U-test, P-value$= 0.057$). Clearly, the mean payoffs of selfish behaviours are lowest in both treatment groups and control groups.

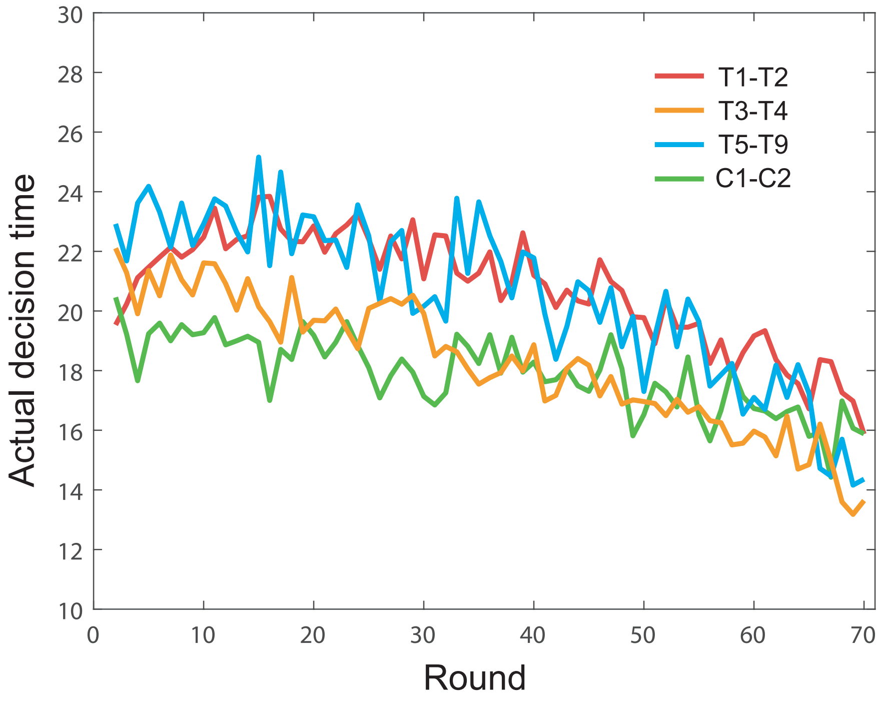

Supplementary Figure 14: Time evolution of actual decision time. Mean actual decision time from round 2 to round 70 in the four categories. Overall, the decision time decreases over rounds. Furthermore, the mean actual decision time in T3-T4 is 3 seconds shorter than T1-T2 and T5-T9, but there is no large difference between T3-T4 and C1-C2.

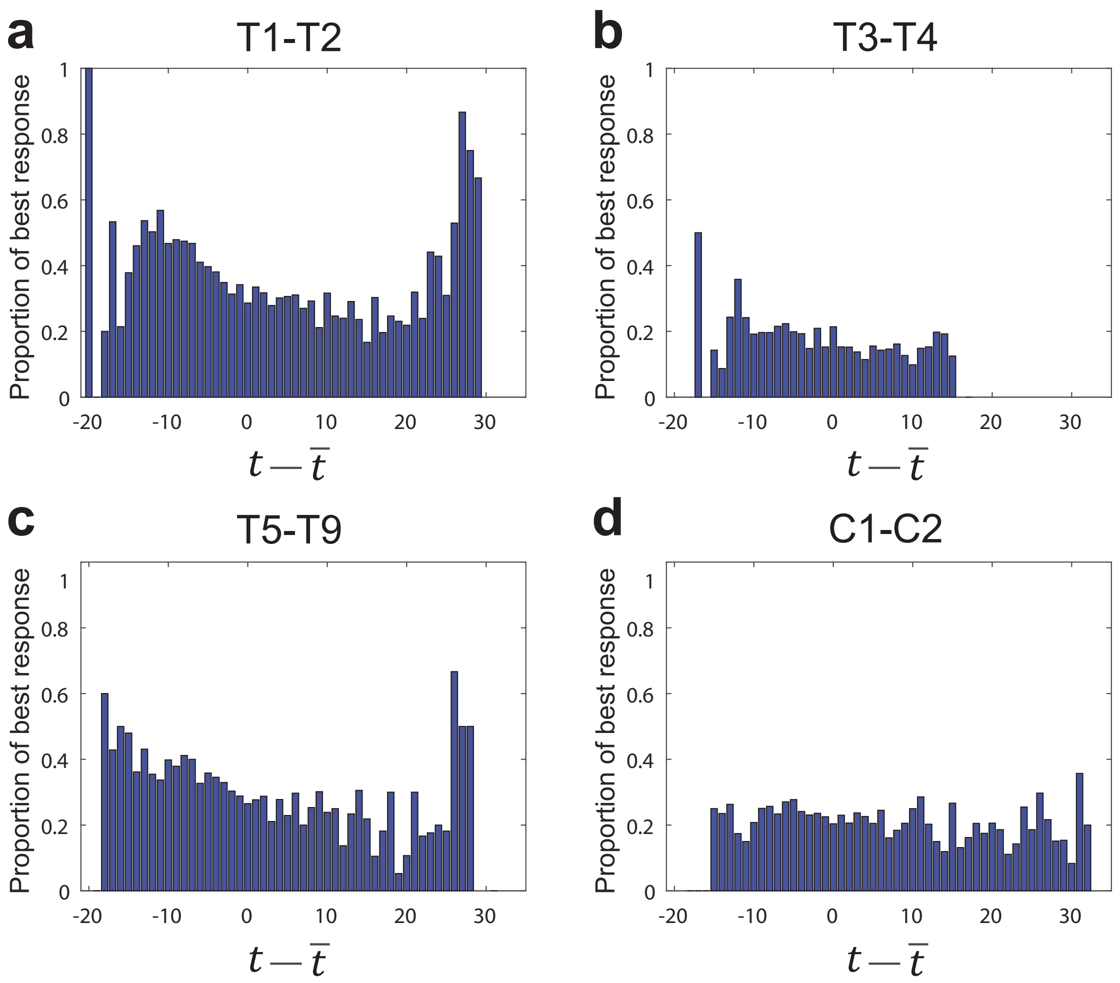

Supplementary Figure 15: Relationship between best-response behaviours and relative decision time. t denotes the actual decision time and $\bar{t}$ is the mean actual decision time in that round. In T1-T2 (a) and T5-T9 (c), we find there are U-shaped relationships between the proportion of best response and $t-\bar{t}$, i.e., both faster and slower decisions are more likely to be best-response. However, in T3-T4 (b), subjects do not have enough time to make a slow decision given the 30 seconds time limit. This explains why the proportion of best-response behaviours in T3-T4 is lower than T1-T2 and T5-T9. Finally, in C1-C2 (d), the correlation between best-response behaviours and $t - \bar{(t)}$ is not significant (Pearson correlation coefficient= 0.0981, P-value= 0.4933). This implies that in decision time may not affect best-response behaviours in a well-mixed population.

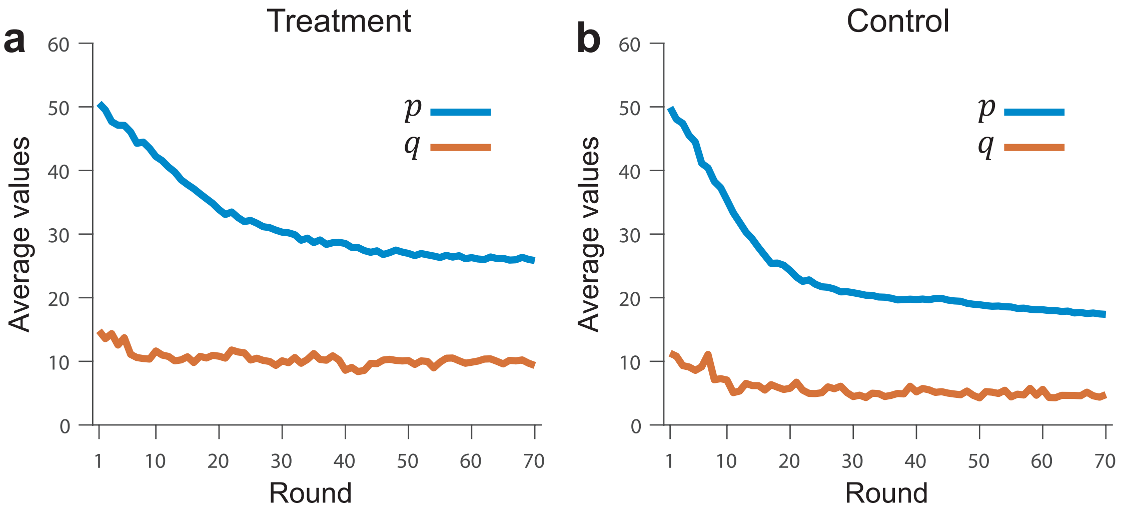

Supplementary Figure 16: Reinforcement learning simulation results. **a**, Mean acceptance levels q are calculated by randomly picking from the treatment database. Mean offers p are reproduced by using reinforcement learning model with fixed interaction structures. **b**, Mean acceptance levels q are calculated by randomly picking from the control database. Mean offers p are reproduced by using reinforcement learning model with well-mixed populations. The results are obtained by averaging 100 independent simulations.

3 Supplementary Tables

Supplementary Table 1: Details of experimental conditions. Our dual-role experiments include four conditions: two large treatment groups (T1 and T2) with structured populations, two median treatment groups (T3 and T4) with structured populations, five small treatment groups (T5-T9) with structured populations, and two control groups (C1 and C2) with well-mixed populations. In our analyses, the data of two subjects in C1 and one subject in T3 are excluded, thus there are only 48 subjects in C2 and 29 subjects in T3 to be analyzed.

	T1	T2	T3-T4	T5-T9	C1	C2
Number of subjects per group	50	53	30	10	50	58
Number of rounds	70	80	100	70	70	80
Decision time per round (sec)	45	45	30	45	45	45
Average income (Yuan)	94	103	119	94	95	104

Supplementary Table 2: A comparison between the theoretical predictions and empirical results Both the single-role and dual-role UG have the same (subgame perfct) Nash equilibrium. Single role MAO UG experiments found $(p, q) \approx (40, 30)$ no matter the population is well-mixed or structured [3]. Most theoretical studies based on evolutionary game theory considered dual-role MAO UG, and found that fixed interaction structures can promote fairness [6, 7].

	Well-mixed	Structured
Single-role Nash equilibrium	$(p, q) = (0, 0)$	$(p, q) = (0, 0)$
Single-role experiments	$(p, q) \approx (40, 30)$	$(p, q) \approx (40, 30)$
Dual-role Nash equilibrium	$(p, q) = (0, 0)$	$(p, q) = (0, 0)$
**Our dual-role experiments**	$(p, q) \approx (15, 5)$	$(p, q) \approx (20, 10)$
Dual-role evolutionary game model	$(p, q) = (0, 0)$	$(p, q) \approx (30, 30)$

Supplementary Table 3: Statistics results for decision time. Mean actual decision time in T1-T2 ($n = 103$), T3-T4 ($n = 59$), T5-T9 ($n = 50$) and C1-C2 ($n = 106$) from round 2 to round 70. Corr represents the correlation coefficient between actual decision time and rounds. The symbol "*" denotes that the correlation is strong, i.e. P-value<0.05.

	Mean	Corr
T1-T2	20.80	-0.8035^*
T3-T4	17.90	-0.7822^*
T5-T9	20.51	-0.8683^*
C1-C2	18.28	-0.9480^*

Supplementary Table 4: The mean values and standard deviations of offers and acceptance levels. We calculate mean values and standard deviations of p and q for all 70 rounds, and separately for rounds 1 to 35 and rounds 36 to 70. Mean(p) and std(p) represent the mean value and the standard deviation of offers of all proposers, respectively, in which a proposer's offer p is taken as the average of his/her offers p over 1-70 rounds/ 1-35 rounds/ 36-70 rounds. Similarly, mean(q) and std(q) represent the mean value and the standard deviation of acceptance levels q of all responders, respectively, in which a responder's acceptance level is taken as the average of his/her acceptance levels q over 1-70 rounds/ 1-35 rounds/ 36-70 rounds.

	1-70 rounds/ 1-35 rounds/ 36-70 rounds			
	mean(p)	std(p)	mean(q)	std(q)
T1-T2	21.66/25.21/18.10	7.11/10.91/5.91	9.86/10.47/9.25	7.04/9.49/7.37
T3-T4	26.14/31.01/21.27	12.93/14.82/12.55	11.90/13.18/10.63	10.87/12.79/11.00
T5-T9	21.12/23.30/18.94	6.67/8.08/6.36	9.97/9.50/10.44	6.51/6.71/7.29
C1-C2	17.78/22.52/13.03	5.40/8.65/3.27	5.70/6.50/4.90	5.82/7.35/5.32

Supplementary Table 5: Mann-Whitney U-test for offer p and acceptance level q. Statistics results of Mann-Whitney U-test for offer p and acceptance level q ($n = 103$ in T1-T2, $n = 59$ in T3-T4, $n = 50$ in T5-T9, and $n = 106$ in C1-C2). A subject's offer (or acceptance level) is taken as the average of his/her p (or q) over 70 rounds. The symbol "*" denotes that the mean values of two groups are significantly different, i.e. P-value<0.05.

P-value for p	T1-T2	T3-T4	T5-T9	C1-C2
T1-T2	1	0.0288*	0.9133	< 0.001*
T3-T4	0.0288*	1	0.0468*	< 0.001*
T5-T9	0.9133	0.0468*	1	< 0.001*
C1-C2	< 0.001*	< 0.001*	< 0.001*	1
P-value for q	T1-T2	T3-T4	T5-T9	C1-C2
T1-T2	1	0.7356	0.8641	< 0.001*
T3-T4	0.7356	1	0.9806	< 0.001*
T5-T9	0.8641	0.9806	1	< 0.001*
C1-C2	< 0.001*	< 0.001*	< 0.001*	1

Supplementary Table 6: Mann-Whitney U-test for proportion of best-response behaviours. Statistics results of Mann-Whitney U-test for proportion of best-response behaviours ($n = 103$ in T1-T2, $n = 59$ in T3-T4, $n = 50$ in T5-T9, and $n = 106$ in C1-C2). The symbol "*" denotes that the mean values of two groups are significantly different, i.e. P-value<0.05.

P-value for p	T1-T2	T3-T4	T5-T9	C1-C2
T1-T2	1	< 0.001*	0.1182	< 0.001*
T3-T4	< 0.001*	1	< 0.001*	0.0028*
T5-T9	0.1182	< 0.001*	1	0.003*
C1-C2	< 0.001*	0.0028*	0.003*	1

4 Supplementary References

[S1]. Ellison G. Learning, local interaction, and coordination. *Econometrica* **61**, 1047–1071 (1993).

[S2]. Brenner T., Vriend N. J. On the behavior of proposers in ultimatum games. *J. Econ. Behav. Organ.* **61**, 617–631 (2006).

[S3]. Han, X. *et al.* Emergence of Communities and Diversity in Social Networks. Natl. Acad. Sci. USA. **114**, 2887–2891. (2017)

[S4]. Fischbacher U. z-Tree: Zurich toolbox for ready-made economic experiments. *Exper. Econ.* **10**, 171–178 (2007).

[S5]. Sutton R. S. & Barto A. G. *Reinforcement learning: An introduction.* (Cambridge: MIT press, 1998).

[S6]. Sinatra, R. *et al.* The ultimatum game in complex networks. *J. Stat. Mech. Theory Exp.* **2009**, P09012 (2009).

[S7]. Iranzo, J., Floría, L. M., Moreno, Y. & Sánchez, A. Empathy emerges spontaneously in the ultimatum game: small groups and networks. *PLoS ONE* **7**, e43781 (2012).
